# Efficacy and tolerability of immunotherapy in advanced nasopharyngeal carcinoma with or without chemotherapy: a meta-analysis

**DOI:** 10.1016/j.bjorl.2021.04.002

**Published:** 2021-04-27

**Authors:** Lifeng Xiao, Wenyi Kang, Jiayu Liao, Yuru Li

**Affiliations:** Southern University of Science and Technology Hospital, E.N.T. Department, Shenzhen, China

**Keywords:** Nasopharyngeal carcinoma, Programmed death-1, Chemotherapy, Immunotherapy, Immune checkpoint inhibitor

## Abstract

**Introduction:**

Evidence of programmed death-1 inhibitors in nasopharyngeal carcinoma has been accumulated. However, previous clinical studies were basically small sample size.

**Objective:**

This study aimed to summarize existing studies to comprehensively compare programmed death-1 inhibitors in nasopharyngeal carcinoma with or without chemotherapy.

**Methods:**

Different databases were searched for full-text publications with a programmed death-1 inhibitor with or without chemotherapy. No study-to-study heterogeneity was detected, and fixed-effect models were applied to synthesize data.

**Results:**

Seven studies were included. The mean progression-free survival duration of programmed death-1 inhibitors treatment was 4.66 months. The 6 month progression-free survival rate was 50%, however, the12 month progression-free survival rate fell to 27%. Comparing with programmed death-1 inhibitor monotherapy, the objective response rate was higher in combination therapy (pooled RR = 2.90, 95% CI: 2.07–4.08). The partial response rate was higher in patients receiving programmed death-1 in association with chemotherapy (pooled RR = 3.09, 95% CI: 2.15–4.46), In contrast, the progressive disease rate was lower in combination therapy group (pooled RR = 0.06, 95% CI: 0.01–0.31). Stable disease condition was comparable (pooled RR = 0.90, 95% CI: 0.50–1.64) with or without chemotherapy. Programmed death-1 single use or combined with chemotherapy did not influence the total adverse events occurrence (pooled RR = 0.99, 95% CI: 0.93–1.05). However, combination therapy could increase the risk of serious adverse events such as anemia, thrombocytopenia, and neutropenia.

**Conclusion:**

The present study summarized the efficacy and safety of programmed death-1 inhibitors in nasopharyngeal carcinoma. Combination therapy showed higher anti-tumor activity except for higher risk of myelosuppression.

## Highlights

• This study aimed to summarize existing studies to comprehensively compare programmed death-1, inhibitors, in nasopharyngeal carcinoma with or without chemotherapy.
• Regarding the efficacy and safety of programmed death-1 inhibitors in nasopharyngeal carcinoma, combination therapy showed higher anti-tumor activity except for higher risk of myelosuppression.
• The incidence of adverse events in programmed death-1 inhibitors-treated patients with any grade showed no difference with or without chemotherapy, except for higher risk of myelosuppression.

## Introduction

Nasopharyngeal carcinoma (NPC) has obvious geographical distribution characteristics, especially in East and Southeast Asia,^1^ and the immune checkpoint inhibitor (ICI) therapy has made a breakthrough in the treatment of recurrent or metastatic diseases of NPC.^2^, ^3^ Immune checkpoint is a kind of immunosuppressive molecules which play an important role of the development of malignant tumors.^4^, ^5^, ^6^ Immune checkpoint has been proved to be effective targets to suppress tumor cells.^7^, ^8^ The discovery of immune checkpoint such as cytotoxic T-lymphocyte-associated protein-4 (CTLA-4),^9^ programmed cell death 1-ligand 1 (PD-L1),^10^, ^11^ and programmed death-1 (PD-1)^10^, ^11^ is of great significance for the development of tumor immunotherapy. In recent years, the immunotherapy represented by PD-1/PD-L1 immune checkpoint inhibitors has changed the current situation of anti-tumor treatment in NPC.^2^, ^12^ A two-arm study total of 67 patients with recurrent and metastatic NPC were sorted into anti-PD1 inhibitor and chemotherapy group and anti-PD1 inhibitor only group, and reported that adding chemotherapy to anti-PD1 inhibitor significantly improved 6 month PFS and OS for NPC patients.^13^ A phase 1/2, open-label, non-comparative study aiming to investigate the safety and anti-tumor activity of PD-1 inhibitor (tislelizumab) in solid tumor showed that the most common adverse event was anemia and increased aspartate aminotransferase. Anti-tumor responses were observed in NPC patients’ subgroup.^14^ The efficacy of nivolumab, another anti-PD-1 inhibitor, has been proved in patients with several types of recurrent and metastatic squamous cell carcinoma of solid tumor.^15^ Sato et al.,^16^ reported that NPC patients enrolled from multiple institutions presented high 1 year survival rate of 75.8%, indicating that nivolumab is a useful and relatively safe second-line systemic therapy in NPC patients. Further evidence of nivolumab came from study reported by Ma et al.^17^ Their phase I/II study investigated the safety and pharmacokinetics of nivolumab in a Chinese NPC cohort. Treatment-related adverse events with Grade 1–2 occurred in 76% patients. This study indicated that nivolumab at 3 mg/kg and flat doses of 240 mg and 360 mg were well tolerated in the NPC patient, and the efficacy showed that nivolumab had promising anti-tumor activity in advanced NPC. Additional study concerning the anti-tumor activity of nivolumab in NPC patients showed that patients with multiply pretreated recurrent or metastatic NPC treating with nivolumab until disease progression attained overall ORR of 20.5%. In addition, this study showed that the proportion of patients who responded was higher among those with PD-L1 positive tumors than those with PD-L1-negative tumors by a descriptive analysis, suggesting nivolumab has promising anti-tumor activity in NPC and hypothesizing local tissue PD-L1 expression as a predictor of nivolumab treatment response.^18^ Fang et al.,^19^ reported a two-arm phase 1 study, with histologically or cytologically confirmed NPC patients receiving camrelizumab monotherapy or combination therapy (camrelizumab + chemotherapy) included. Camrelizumab as one of the anti-PD-1 inhibitors was regarded as a well-tolerated and potential treatment option for patients with recurrent or metastatic NPC. Further, the toxicity of the combination of camrelizumab plus gemcitabine and cisplatin was manageable, and the anti-tumor activity of combination therapy was promising. The safety profile and antitumor activity of pembrolizumab (Keytruda), a humanized monoclonal anti-PD1 antibody were verified in patients with PD-L1-positive advanced solid tumors. Moreover, the NPC cohort in this study included unresectable or metastatic disease validated the anti-tumor activity and a manageable safety profile issues of pembrolizumab in twenty-seven NPC patients receiving monotherapy.^20^

As is shown in abovementioned clinical studies, evidence of anti-PD-1 treatment in NPC has been accumulated. However, previous clinical studies were basically small sample-sized. Besides, contradictory results were even acquired. This study aimed to summarize existed studies to generally interpret the survival condition of NPC patients. Besides, this study was designed to comprehensively compare the efficacy and safety of PD-1 inhibitors in NPC with or without chemotherapy as combination therapy.

## Methods

### Search strategy

We conducted this meta-analysis of the current literatures according to the Preferred Reporting Items for Systematic Reviews and Meta-Analyses (PRISMA) guidelines. A comprehensive systematic search of several major electronic databases (PubMed, Embase, Web of Science, Ovid, EBSCO, clinicaltrials.gov and the Cochrane library) was conducted before February 1st, 2021. The following search terms used were nasopharyngeal carcinoma, immunotherapy, immune checkpoint inhibitor, cytotoxic T-lymphocyte antigen-4 or CTLA-4, anti-Programmed Death-1 or anti-PD-1, programmed death ligand-1, PD-L1 or anti-PD-L1. No language restriction and publication status were imposed. Additional relevant articles were obtained by searching the reference lists of the articles included in this study.

### Study selection criteria and data extraction

Two investigators independently performed the literature search in accordance with the inclusion criteria and exclusion criteria. Disagreements were resolved through discussion and consensus or solved by the third investigator. Studies that met the following inclusive criteria were considered eligible for this meta-analysis: (1) Study design: cohort study; (2) Population: advanced nasopharyngeal carcinoma; (3) Study intervention: immune checkpoint inhibitor in combination with standard chemotherapy; (4) PD-1 inhibitors single use or in combination with standard chemotherapy; (5) Outcome measures: OS, PFS, ORR, and adverse events.

In cases of different publications from the same study, the one with the complete data was chosen. Interesting data such as the number of total patients and the number of patients with clearly defined events should be carefully collected. In addition, baseline demographic data and follow-up duration were extracted. Data of each parameter available for meta-analysis was extracted, including mean progression-free survival (PFS) duration, 6-month overall survival (6mOS) rate, 12-month overall survival (12mOS) rate, 6-month progression-free survival (6mPFS) rate, 12-month progression-free survival (12mPFS) rate, objective response rate (ORR), disease control rate (DCR), complete response (CR) rate, partial response (PR) rate, stable disease (SD) rate, progressive disease (PD) rate, any adverse events, abnormal liver function with any grade, hypothyroidism with any grade, and anemia with any grade in single-arm study. For double-arm study, parameters of ORR, CR, PR, SD, PD, any adverse events, abnormal liver function with any grade, hypothyroidism with any grade, anemia with grade ≥3, thrombocytopenia with grade ≥3, and neutropenia with grade ≥3 were collected.

### Data synthesis and analysis

Endnote (Version 7.6, Thomson Reuters, Inc., Philadelphia, PA) bibliographic software was used to create an electronic library of citations identified in the database searches. PubMed searches were performed using Endnote, and duplicate records were deleted. Each study was assigned a unique identification code to enable tracking of reviews and analysis after title/abstract screening. Two independent investigators used a standardized tool to extract the following data from each study: first author's name, year of publication, country, number of study patients, baseline patient characteristics (age, sex, race, diabetes duration), and outcome measures. We used the recommended method to assess the risk of bias for included studies, and the specific items were shown in the Supplemental Table 1.

### Statistics

Data were analyzed using Stata version 12.0 (Stata Corporation, College Station, TX, USA). Before the data were synthesized, we first test the heterogeneity between the studies using *I*^2^ statistic to describe the percentage of the variability that attributed to heterogeneity across the studies. Studies with an *I*^2^ statistic of <50% was considered to have low degree of heterogeneity. Pooled estimates were calculated using a fixed-effects model (Mantel-Haenszel method); otherwise, a random-effects model (DerSimonian-Laird method) was applied when significant heterogeneity among the included studies was found. Dichotomous variables were expressed as Relative Risk (RR) with 95% Confidence Interval (95% CI). The assessment of publication bias was evaluated by using Egger and Begger test. A 2-tailed *p*-value less than 0.05 was judged as statistically significant, except where otherwise specified.

## Results

### Literature search and general description of included studies

**Figure 1:**
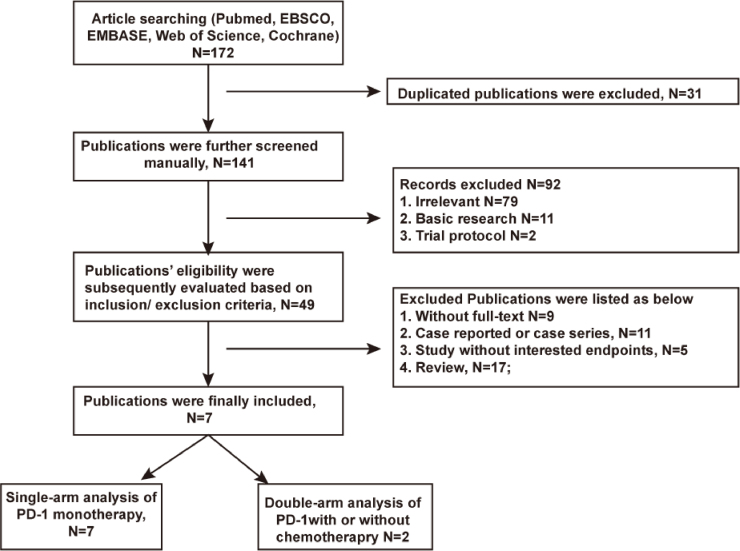
Publication filtration.

A total of 172 articles was searched after excluding 31 duplications. 42 articles were excluded afterward for not meeting the inclusion/exclusion criteria. Seven articles^13^, ^14^, ^16^, ^17^, ^18^, ^19^, ^20^ finally passed the eligibility assessment. The flow diagram of publication filtration was shown in Fig. 1. Data of 296 patients with follow-up duration for as long as 48-months were pooled together, and the characteristics of included studies were depicted in the Table 1. Characteristics of patients’ demography at baseline were described in the Table 2. The assessment of literature quality was shown in the Supplemental Table 1 indicating the compatibility of included studies.

**Table 1:** Characteristics of included studies.

Author	Year	Region	N° of patients	Anti PD-1 drug	Study design	Mean follow-up duration
Jin, et al.	2021	China Mainland	67	Camrelizumab, Toripalimab, Penpulimab, Tislelizumab	Prospective cohort study	7-months
Sato, et al.	2020	Japan	12	Nivolumab	Prospective cohort study	11.9-months
Shen, et al.	2020	China Mainland	300	Tislelizumab	Prospective cohort study	8.1-months
Ma, et al.	2019	China Mainland	32	Nivolumab	Prospective cohort study	7.5-months
Fang, et al.	2018	China Mainland	93	Camrelizumab	Prospective cohort study	10-months
Ma, et al.	2018	China Hong Kong district	44	Nivolumab	Prospective cohort study	12.5-months
Hsu, et al.	2017	China Taiwan district	27	Pembrolizumab	Prospective cohort study	20-months

**Table 2:** Characteristics of patients’ demography at baseline.

Author	Age, year (range)	Male (%)	N of ECOG PS ≤1 (%)	Local recurrence (%)	Metastasis (%)	N of prior lines of chemotherapy ≤2 (%)	Median study follow-up duration, months (range)	Dosage of anti PD-1 drugs
Jin et al., 2021	N of Age ≥60-year (%) 22 (32.8)	50 (74.6)	57 (85.1)	12 (17.9)	55 (82.1)	57 (85.1)	7 (2–19)	Camrelizumab: 200-mg Q2W; Toripalimab: 240-mg Q3W; Penpulimab: 200-mg Q2W; Tislelizumab: 200-mg Q3W
Sato et al., 2020	58 (30–67)	10 (83)	12 (100)	8 (67)	12 (100)	7 (58)	11.9 (2.8–21.7)	Nivolumab: 3-mg/kg Q2W; 240-mg Q2W
Shen et al., 2020	56.5 (18–82)	207 (69)	300 (100)	NG	284 (95)	158 (55)	8.1 (0.2–21.9)	Tislelizumab: 200-mg Q3W
Ma et al., 2019	48 (27–72)	31 (67.4)	46 (100)	NG	NG	NG	7.5 (0.8–24.7)	Nivolumab: 3-mg/kg Q2W; 240-mg Q2W; 360-mg Q3W
Fang et al., 2018	45 (38–52)	75 (81)	93 (100)	4 (4)	89 (95)	59 (64)	9.9 (8.1–11.7)	Camrelizumab: 200-mg Q2W
Ma et al., 2018	57 (37–76)	35 (77.8)	44 (97.8)	13 (29.5)	32 (70.5)	17 (38.6)	9.3 (3.6–13.1)	Nivolumab: 3-mg/kg Q2W
Hsu et al., 2017	52 (18–68)	21 (77.8)	27 (100)	9 (33.1)	18 (66.9)	8 (29.6)	20 (2.2–26.8)	Pembrolizumab: 10-mg/kg Q2W

PD-1, Programmed Death-1; NG, Not Given; N, number; ECOG, Eastern Cooperative Oncology Group; PS, Performance Status.

### Analysis of the efficacy of PD-1 treatment

**Figure 2:**
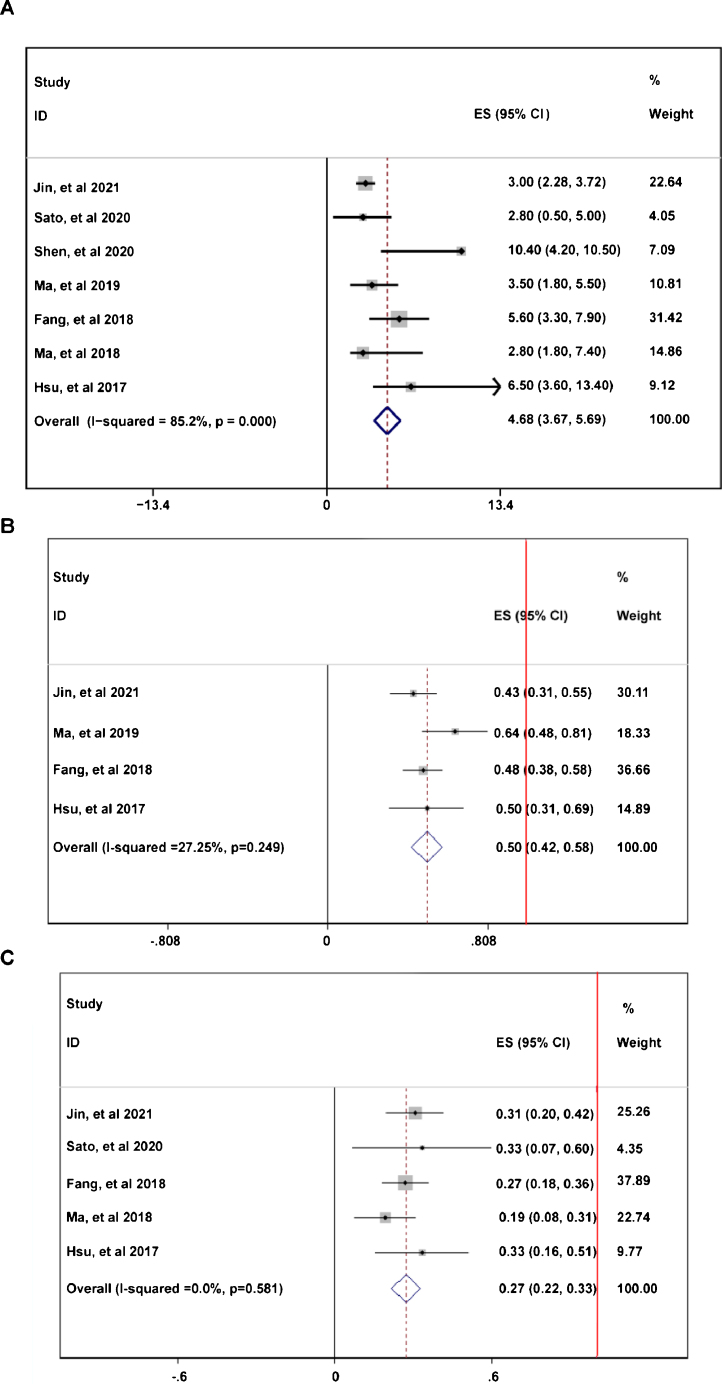
The Progression Free Survival (PFS) of patients receiving PD-1 treatment: mean PFS (A), 6-month PFS rate (B), and 12-month PFS rate. The red vertical line was presented as the reference line of *x* = 1.

**Figure 3:**
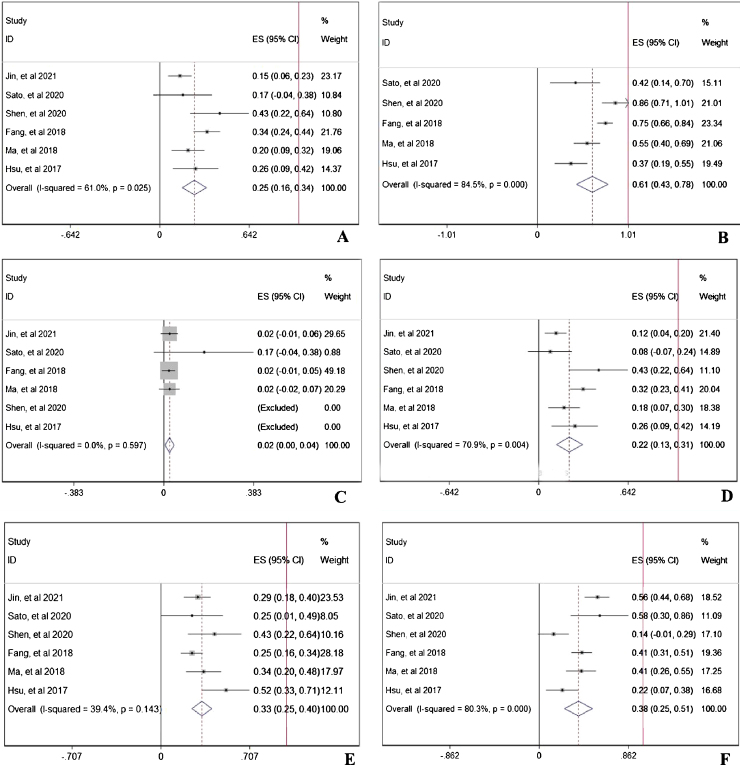
The evaluation of disease condition in patients receiving PD-1 treatment: objective response rate (A), disease control rate (B), complete response rate (C), partial response rate (D), stable disease rate (E), and progressive disease rate (F). The red vertical line was presented as the reference line of *x* = 1.

During the follow-up duration, the progression free survival (PFS) parameters of patients receiving PD-1 treatment: mean PFS duration (A), 6-month PFS rate (B), and 12-month PFS rate were reported in 7 articles without heterogeneity (*I*^2^ < 50%).^13^, ^14^, ^16^, ^17^, ^18^, ^19^, ^20^ The pooled data indicated that the mean PFS duration of patients who receiving PD-1 inhibitors treatment was 4.66-month with 95% confidential interval (CI): 3.76–5.69 month (Fig. 2A). Overall 6-month PFS rate in included patients was 50% with 95% CI between 42% to 58% (Fig. 2B). However, 12-month PFS rate fell to 27% with 95% CI: 22–33% (Fig. 2C). To evaluate the disease condition in patients receiving PD-1 treatment, rates of ORR,^13^, ^14^, ^16^, ^18^, ^19^, ^20^ DCR,^14^, ^16^, ^18^, ^19^, ^20^ CR,^13^, ^14^, ^16^, ^18^, ^19^, ^20^ PR,^13^, ^14^, ^16^, ^18^, ^19^, ^20^ SD,^13^, ^14^, ^16^, ^18^, ^19^, ^20^ and PD^13^, ^14^, ^16^, ^18^, ^19^, ^20^ were analyzed. The pooled ORR indicating the combination of CR rate and PR rate was 25% (Fig. 3A). The pooled DCR, including 3 disease condition: CR, PR, and SD was 61% (Fig. 3B). The pooled PR rate was 22% (Fig. 3D), however, the pooled CR rate was 2% (Fig. 3C). The pooled SD rate was 33% (Fig. 3E), and the pooled PD rate was 38% (Fig. 3F).

### Analysis of the PD-1 treatment related adverse events

**Figure 4:**
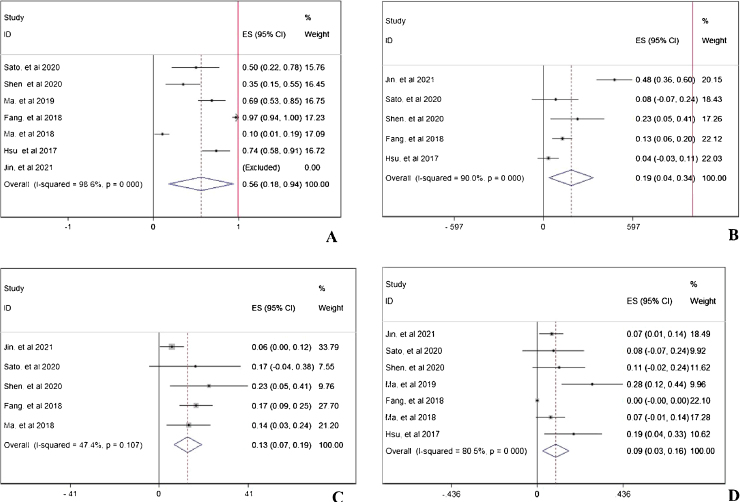
The PD-1 treatment related adverse events: any adverse events (A), anemia (B), abnormal liver function (C), and hypothyroidism (D). The red vertical line was presented as the reference line of *x* = 1.

In prospective view, the occurrence of total adverse events with any grade^13^, ^14^, ^16^, ^17^, ^18^, ^19^, ^20^ in PD-1 treated patients was 56% with 95% CI: 18–94% (Fig. 4A). To further investigate the three most important adverse events, incidences of anemia,^13^, ^14^, ^16^, ^19^, ^20^ abnormal liver function,^13^, ^14^, ^16^, ^18^, ^19^ and hypothyroidism^13^, ^14^, ^16^, ^17^, ^18^, ^19^, ^20^ were analyzed respectively. The pooled occurrence rates of anemia, abnormal liver function, and hypothyroidism were 19% (Fig. 4B), 13% (Fig. 4C), and 9% (Fig. 4D).

### Double-arm analysis of PD-1 efficacy: with or without chemotherapy

**Figure 5:**
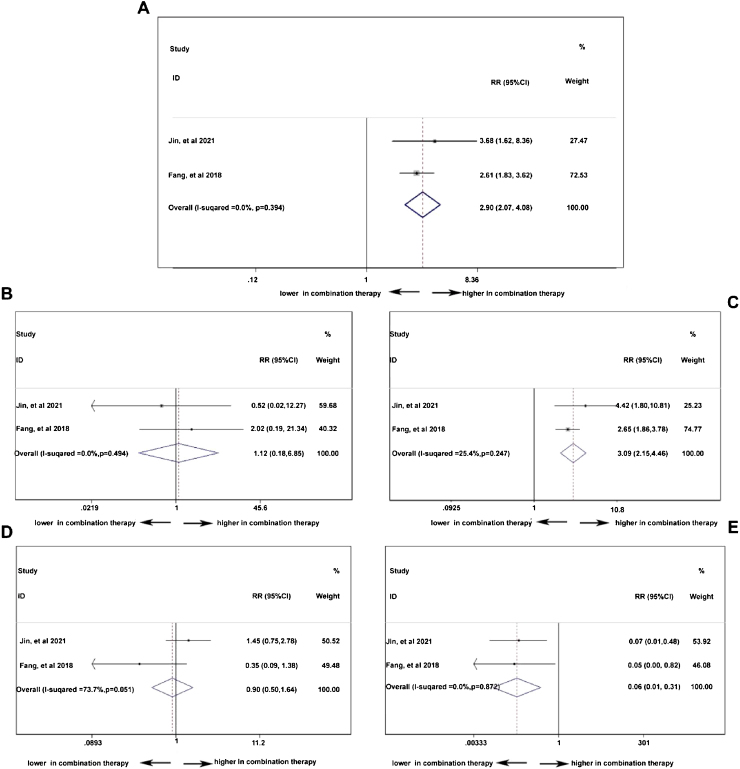
The efficacy of PD-1 treatment with or without chemotherapy: objective response rate (A), complete response rate (B), partial response rate (C), stable disease rate (D), and progressive disease rate (E).

To compare the difference of treatment response between PD-1 monotherapy versus PD-1 in combination with standard chemotherapy in NPC patients, parameters such as ORR, CR, PR, SD, and PD^13^, ^19^ were analyzed. In Fig. 5A, the ORR was higher in patients with additional chemotherapy to PD-1 treatment (pooled RR = 2.90, 95% CI: 2.07–4.08). However, in Fig. 5B, the CR rate showed no difference no matter the existence of additional chemotherapy to PD-1 treatment (pooled RR = 1.12, 95% CI: 0.18–6.85). The PR rate was higher in patients receiving PD-1 in alliance with chemotherapy (pooled RR = 3.09, 95% CI: 2.15–4.46), indicating cooperative effect of PD-1 and standard chemotherapy (Fig. 5C). Conversely, the PD rate was lower in combination therapy group (pooled RR = 0.06, 95% CI: 0.01–0.31), which is in accordance with the result in increased PR rate (Fig. 5E). Stable condition of NPC disease was comparable (pooled RR = 0.90, 95% CI: 0.50–1.64) with or without chemotherapy (Fig. 5D).

### Double-arm analysis of PD-1 safety: with or without chemotherapy

**Figure 6:**
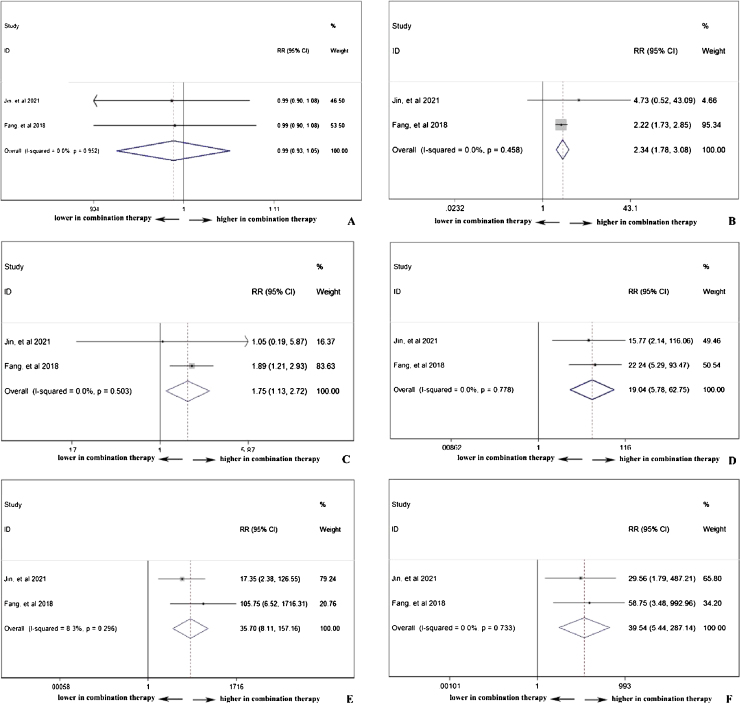
The safety of PD-1 treatment with or without chemotherapy: any adverse events (A), bbnormal liver function with any grade (B), hypothyroidism (C), Anemia with grade ≥3 (D), neutropenia with grade ≥3 (E), and thrombocytopenia with grade ≥3 (F).

PD-1 single use or combined with chemotherapy did not influence the total adverse events occurrence (pooled RR = 0.99, 95% CI: 0.93–1.05), indicating chemotherapy in addition to PD-1 adding no extra risk of medication side effects (Fig. 6A). However, in Fig. 6B, the combination therapy could increase the risk of abnormal liver function with any grade (pooled RR = 2.34, 95% CI: 1.78–3.08), indicating cooperative effect of PD-1 and chemotherapy. PD-1 was proved to negatively influence thyroid function, and as shown in Fig. 6C, the combination of PD-1 and chemotherapy significantly increased the risk of hypothyroidism incidence (pooled RR = 1.75, 95% CI: 1.13–2.72). Considering serious adverse events (grade ≥3), anemia (Fig. 6D), thrombocytopenia (Fig. 6E), and neutropenia (Fig. 6F) were further investigated. The occurrence rate of anemia (pooled RR = 19.04, 95% CI: 5.78–62.75), thrombocytopenia (pooled RR = 35.70, 95% CI: 8.11–157.16), and neutropenia (pooled RR = 39.54, 95% CI: 5.44–287.14) with grade ≥3 was far higher in combination therapy group, indicating that patients who received both PD-1 inhibitors and chemotherapy were susceptible to myelosuppression.

### Publication bias

Publication bias analyzed by Begg's test showed a symmetrical distribution of included publications (*p* = 0.707) in a funnel plot (Supplemental Fig. 1), and this indicated that there did not exist publication bias among articles included in the present study.

## Discussion

Clinical evidence of PD-1 inhibitors in NPC patients have been accumulated. However, previous clinical studies were basically characterized by a small sample size. This study summarized existing studies to generally interpret the survival condition of NPC patients, and was designed to comprehensively compare the efficacy and safety of PD-1 inhibitors in NPC patients with or without additional application of standard chemotherapy.

Our analysis covered seven independent studies ranging from 2017 to 2021. Data of 296-patients with followup duration for as long as 48-months were pooled together. To our knowledge, this is the first synthetic study to compare the efficacy and safety of PD-1 inhibitors in NPC patients with or without additional application of standard chemotherapy to date. Data from our study indicated the mean PFS duration of PD-1 inhibitors treatment was 4.66-months (95% CI: 3.76–5.69 month). The 6-month PFS rate was 50% (95% CI: 42–58%), however, 12-month PFS rate fell to 27% (95% CI: 22–33%). The occurrence rate of total adverse events with any grade in PD-1 treated patients was 56% (95% CI: 18–94%). Comparing with PD-1 inhibitor monotherapy, the ORR was higher in combination therapy (pooled RR = 2.90, 95% CI: 2.07–4.08). The partial response rate was higher in patients receiving PD-1 in alliance with chemotherapy (pooled RR = 3.09, 95% CI: 2.15–4.46), In contrast, the progressive disease rate was lower in combination therapy group (pooled RR = 0.06, 95% CI: 0.01–0.31). Stable disease condition was comparable (pooled RR = 0.90, 95% CI: 0.50–1.64) with or without chemotherapy. PD-1 single use or combined with chemotherapy did not influence the total adverse events occurrence (pooled RR = 0.99, 95% CI: 0.93–1.05). However, the combination therapy could increase the risk of serious adverse events such as anemia, thrombocytopenia, and neutropenia.

In the past few years, some areas of NPC treatment made some outstanding progression, especially in liquid biopsy, minimally invasive surgery, chemotherapy, and immunotherapy.^21^

Recently, a systemic review presented that the aggregated ORR and drug-related adverse events were favorable and manageable in NPC patients receiving anti-PD-1 treatment.^22^ However, this study failed to perform the head-to-head comparison of PD-1 inhibitors with or without traditional evaluation. Additionally, our investigation updated the clinical data by including more clinical studies. In recent years, advances in immune checkpoint inhibitors therapy significantly improve the therapeutic effects of malignant tumor.^5^, ^9^ The anti-PD-1 antibody showed strong anti-tumor activity in clinical trials concerning on NPC.^12^, ^13^, ^20^ Synergistic effects of PD-1 inhibitors with chemotherapy, radiotherapy, molecular targeted therapy or other kinds of biological therapy were validated in different kinds of cancer.^23^, ^24^, ^25^, ^26^, ^27^ A recent comprehensive analysis showed that induction chemotherapy is superior to concurrent chemotherapy alone for locally advanced nasopharyngeal carcinoma.^28^ However, the effects of induction chemotherapy prior to anti-PD-1 treatment has not been reported, and is worth of further research. For the tolerability and safety concerns, PD-1 inhibitors-based combination therapy could potentially carry an additional risk ranging from slight to severe.^29^, ^30^ Nonetheless, other studies reported that the adverse events after anti-PD-1 treatment showed no difference with or without concomitant chemotherapy.^13^, ^19^, ^22^ As immunotherapy is incorporated into the standard treatment mode, the optimal combination of targeted drugs and immune adjuvants, and the sequence of chemotherapy and radiotherapy will need to be solved. Though PD-1 inhibitors showed an exciting predominance on treating NPC, there were some NPC patients being reported to negatively respond to anti-PD-1 treatment.^31^, ^32^, ^33^ Wang et al., emphasized the prognostic significance of PD-L1 and PD-1 expression in patients with NPC, the expression status immune checkpoints in NPC patients might add prognostic value to the tumor staging system.^34^ Further, the expression of PD-1 and PD-L1 in locoregionally advanced NPC tissue significantly correlated with disease outcome and the response to immunotherapy including efficacy and safety.^32^, ^35^, ^36^, ^37^, ^38^, ^39^ In addition to PD-1/PD-L1 targeted immunotherapy, other targets of NPC have been reported.^40^ NPC is thought to be associated with Epstein Barr Virus (EBV), characterized by peritumoral immune infiltration.^12^ Immunotherapy targeting EBV antigen has been explored in clinical trials;^40^, ^41^, ^42^ it may become an important target for NPC. Besides, cancer vaccine^43^, ^44^, ^45^ and adoptive T-cell^46^, ^47^, ^48^ therapy have been explored in clinical studies.

As far as we know, this was the first meta-analysis to compare the efficacy and safety of PD-1 inhibitors in NPC patients with or without additional application of standard chemotherapy. In the present synthetic study, the efficacy and safety of PD-1 inhibitors in NPC was summarized. Combination therapy showed higher anti-tumor activity except for higher risk of myelosuppression. However, results from this study should be updated in future with more studies with high quality.

## Limitations

This meta-analysis included seven studies, and all of these included publications were of prospective cohort design. However, the included articles were not with randomization control design. Besides, some studies were small in scale. Furthermore, only a few articles were eligible for the criteria of selection, and thus sensitivity analysis was not applicable. Consequently, large scale, prospective, multi-center, and randomized clinical trials are still highly needed with clearly reporting confounding factors.

## Conclusion

The present study summarized existing studies to generally interpret the survival condition of NPC patients and to comprehensively compare PD-1 inhibitors in NPC with or without chemotherapy. From this study, combination therapy of PD-1 inhibitors and chemotherapy showed higher anti-tumor activity. The incidence of adverse events in PD-1 inhibitors treated patients with any grade showed no difference with or without chemotherapy, except for higher risk of myelosuppression.

## Funding

This research did not receive any specific grant from funding agencies in the public, commercial, or not-for-profit sectors.

## Conflicts of interest

The authors declare no conflicts of interest.

## Footnotes

Appendix A Supplementary material related to this article can be found, in the online version, at doi:10.1016/j.bjorl.2021.04.002.
