# Supplementary material for: Efficacy and tolerability of immunotherapy in advanced nasopharyngeal carcinoma with or without chemotherapy: a meta-analysis
Source: Braz J Otorhinolaryngol. 2021 Apr 27;88(Suppl 1):S70–81. doi: 10.1016/j.bjorl.2021.04.002 (PMC9734274; doi:10.1016/j.bjorl.2021.04.002)
Supplement: Supplementary file 1 [file mmc1.docx]

BJORL-D-21-00155_Supplementary material

**Supplemental Figure 1** The publication bias analysis by funnel plots: for single-arm study (A) and for double-arm study (B), respectively.


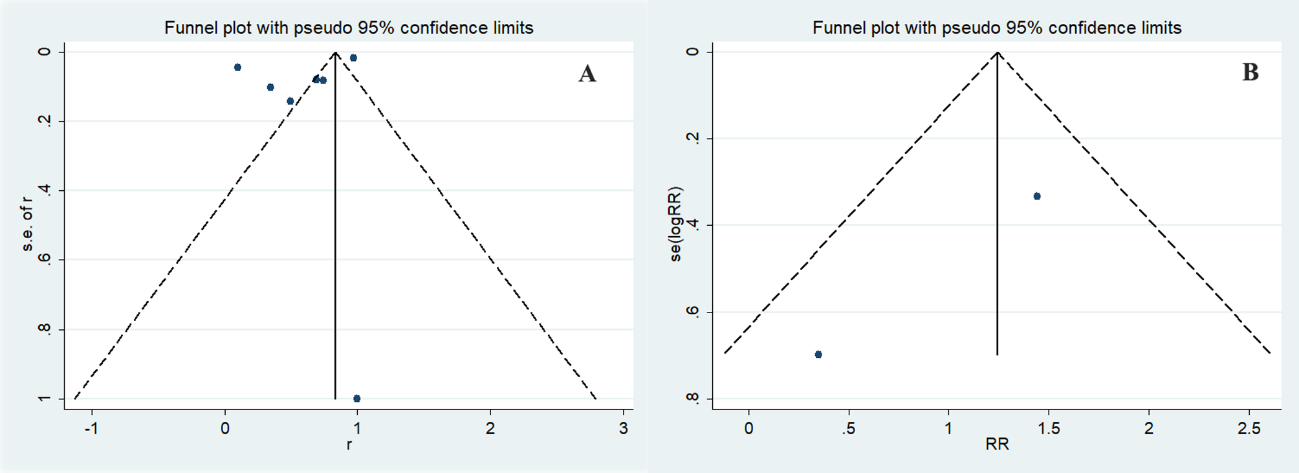


**Supplemental Table 1** Quality assessment of eligible literatures.

| **Author** | **Prospective design** | **Clear definition of study population** | **(1)** | **(2)** | **(3)** | **(4)** | **(5)** |
| --- | --- | --- | --- | --- | --- | --- | --- |
| Jin et al., 2021 | Yes | Yes | Yes | Stable | Yes | Yes | Yes |
| Sato et al., 2020 | Yes | Yes | Yes | Stable | Yes | Yes | Yes |
| Shen et al., 2020 | Yes | Yes | Yes | Stable | Yes | Yes | Yes |
| Ma et al., 2019 | Yes | Yes | Yes | Stable | Yes | Yes | Yes |
| Fang et al., 2018 | Yes | Yes | Yes | Stable | Yes | Yes | Yes |
| Ma et al., 2018 | Yes | Yes | Yes | Stable | Yes | Yes | Yes |
| Hsu et al., 2017 | Yes | Yes | Yes | Stable | Yes | Yes | Yes |

(1) Whether the study design was suitable for the disease condition and treatment; (2) Were the treatment stable or fluctuating; (3) Was different cohort comparable to each other; (4) Was there any clear definition of end event; (5) Was plan of follow-up clearly given.
